# Imperfect Vaccination Can Enhance the Transmission of Highly Virulent Pathogens

**DOI:** 10.1371/journal.pbio.1002198

**Published:** 2015-07-27

**Authors:** Andrew F. Read, Susan J. Baigent, Claire Powers, Lydia B. Kgosana, Luke Blackwell, Lorraine P. Smith, David A. Kennedy, Stephen W. Walkden-Brown, Venugopal K. Nair

**Affiliations:** 1 Center for Infectious Disease Dynamics, Departments of Biology and Entomology, The Pennsylvania State University, University Park, Pennsylvania, United States of America; 2 Fogarty International Center, National Institutes of Health, Bethesda, Maryland, United States of America; 3 Avian Oncogenic Virus Group, The Pirbright Institute, Compton, Newbury, Berkshire, United Kingdom; 4 School of Environmental and Rural Science, The University of New England, Armidale, Australia; Imperial College London, UNITED KINGDOM

## Abstract

Could some vaccines drive the evolution of more virulent pathogens? Conventional wisdom is that natural selection will remove highly lethal pathogens if host death greatly reduces transmission. Vaccines that keep hosts alive but still allow transmission could thus allow very virulent strains to circulate in a population. Here we show experimentally that immunization of chickens against Marek's disease virus enhances the fitness of more virulent strains, making it possible for hyperpathogenic strains to transmit. Immunity elicited by direct vaccination or by maternal vaccination prolongs host survival but does not prevent infection, viral replication or transmission, thus extending the infectious periods of strains otherwise too lethal to persist. Our data show that anti-disease vaccines that do not prevent transmission can create conditions that promote the emergence of pathogen strains that cause more severe disease in unvaccinated hosts.

## Abstract 2

A study using Marek's disease virus in poultry shows that by reducing natural selection against highly virulent strains, imperfect vaccination enables the spread of viral strains that would otherwise be too lethal to persist.

## Author Summary

There is a theoretical expectation that some types of vaccines could prompt the evolution of more virulent (“hotter”) pathogens. This idea follows from the notion that natural selection removes pathogen strains that are so “hot” that they kill their hosts and, therefore, themselves. Vaccines that let the hosts survive but do not prevent the spread of the pathogen relax this selection, allowing the evolution of hotter pathogens to occur. This type of vaccine is often called a leaky vaccine. When vaccines prevent transmission, as is the case for nearly all vaccines used in humans, this type of evolution towards increased virulence is blocked. But when vaccines leak, allowing at least some pathogen transmission, they could create the ecological conditions that would allow hot strains to emerge and persist. This theory proved highly controversial when it was first proposed over a decade ago, but here we report experiments with Marek’s disease virus in poultry that show that modern commercial leaky vaccines can have precisely this effect: they allow the onward transmission of strains otherwise too lethal to persist. Thus, the use of leaky vaccines can facilitate the evolution of pathogen strains that put unvaccinated hosts at greater risk of severe disease. The future challenge is to identify whether there are other types of vaccines used in animals and humans that might also generate these evolutionary risks.

## Introduction

Infectious agents can rapidly evolve in response to health interventions [1]. Here, we ask whether pathogen adaptation to vaccinated hosts can result in the evolution of more virulent pathogens (defined here to mean those that cause more or faster mortality in unvaccinated hosts).

Vaccination could prompt the evolution of more virulent pathogens in the following way. It is usually assumed that the primary force preventing the evolutionary emergence of more virulent strains is that they kill their hosts and, therefore, truncate their own infectious periods. If so, keeping hosts alive with vaccines that reduce disease but do not prevent infection, replication, and transmission (so-called “imperfect” vaccines) could allow more virulent strains to circulate. Natural selection will even favour their circulation if virulent strains have a higher transmission in the absence of host death or are better able to overcome host immunity. Thus, life-saving vaccines have the potential to increase mean disease virulence of a pathogen population (as assayed in unvaccinated hosts) [2–4].

The plausibility of this idea (hereafter called the “imperfect-vaccine hypothesis”) has been confirmed with mathematical models [2,5–9]. Efficacy and mode of action are key. If the vaccine is sterilizing, so that transmission is stopped, no evolution can occur. But if it is non-sterilizing, so that naturally acquired pathogens can transmit from immunized individuals (what we hereafter call a “leaky” vaccine), virulent strains will be able to circulate in situations in which natural selection would have once removed them [2]. Thus, anti-disease vaccines (those reducing in-host replication or pathogenicity) have the potential to generate evolution harmful to human and animal well-being; infection- or transmission-blocking vaccines do not [2–9]. Note that the possibility of vaccine-driven virulence evolution is conceptually distinct from vaccine-driven epitope evolution (antigenic escape), in which variants of target antigens evolve because they enable pathogens that are otherwise less fit to evade vaccine-induced immunity. The evolution of escape variants has been frequently observed [4,10].

The imperfect-vaccine hypothesis attracted controversy [11–14], not least because human vaccines have apparently not caused an increase in the virulence of their target pathogens. But most human vaccines are sterilizing (transmission-blocking) or not in widespread use or only recently introduced [4]. Moreover, unambiguous comparisons of strain virulence and the impact of vaccination on transmission require experimental infections in the natural host—clearly impossible for human diseases. The situation is different for veterinary infections. Here, we report experiments with Marek’s disease virus (MDV), a highly contagious oncogenic herpesvirus that costs the global poultry industry more than $US2 billion annually [15]. We test a key prediction of the imperfect-vaccine hypothesis: that vaccination will elevate the fitness of highly virulent strains above that of less virulent strains.

Chickens become infected with MDV by inhalation of dust contaminated with virus shed from the feather follicles of infected birds. In a contaminated poultry house, chicks are infected soon after hatching and remain infectious for life [16]. The virus can remain infectious in poultry dust for many months [17,18]. As originally described, Marek’s disease (MD) was a paralysis of older birds, but by the 1950s, “acute MD” characterised by lymphomas in multiple organs in younger birds was occurring. This became the dominant form of MD, with increasing virulence, characterised by more severe lymphomas and mortality at increasingly early ages and, under some circumstances, paralysis and death in the first weeks of life, well before lymphoma formation [15,19].

MDV has been evolving in poultry immunized with leaky anti-disease vaccines since the introduction of the first vaccines in 1970 [15,19–24]. All MD vaccines are live viruses administered to 18-day-old embryos or immediately after hatch, and vaccinated birds can become infected and shed wild-type virus [25–28]. Wild-type MD viruses are so-called serotype 1 viruses. First-generation vaccines include a serotype 3 herpesvirus of turkeys called HVT; second-generation vaccines are a combination of HVT and SB-1, a serotype 2 isolate. Third-generation vaccines are based on an attenuated serotype-1 virus isolate CVI998, the so-called Rispens vaccine [15,19–24].

## Results

### Viral Shedding

**Fig 1:**
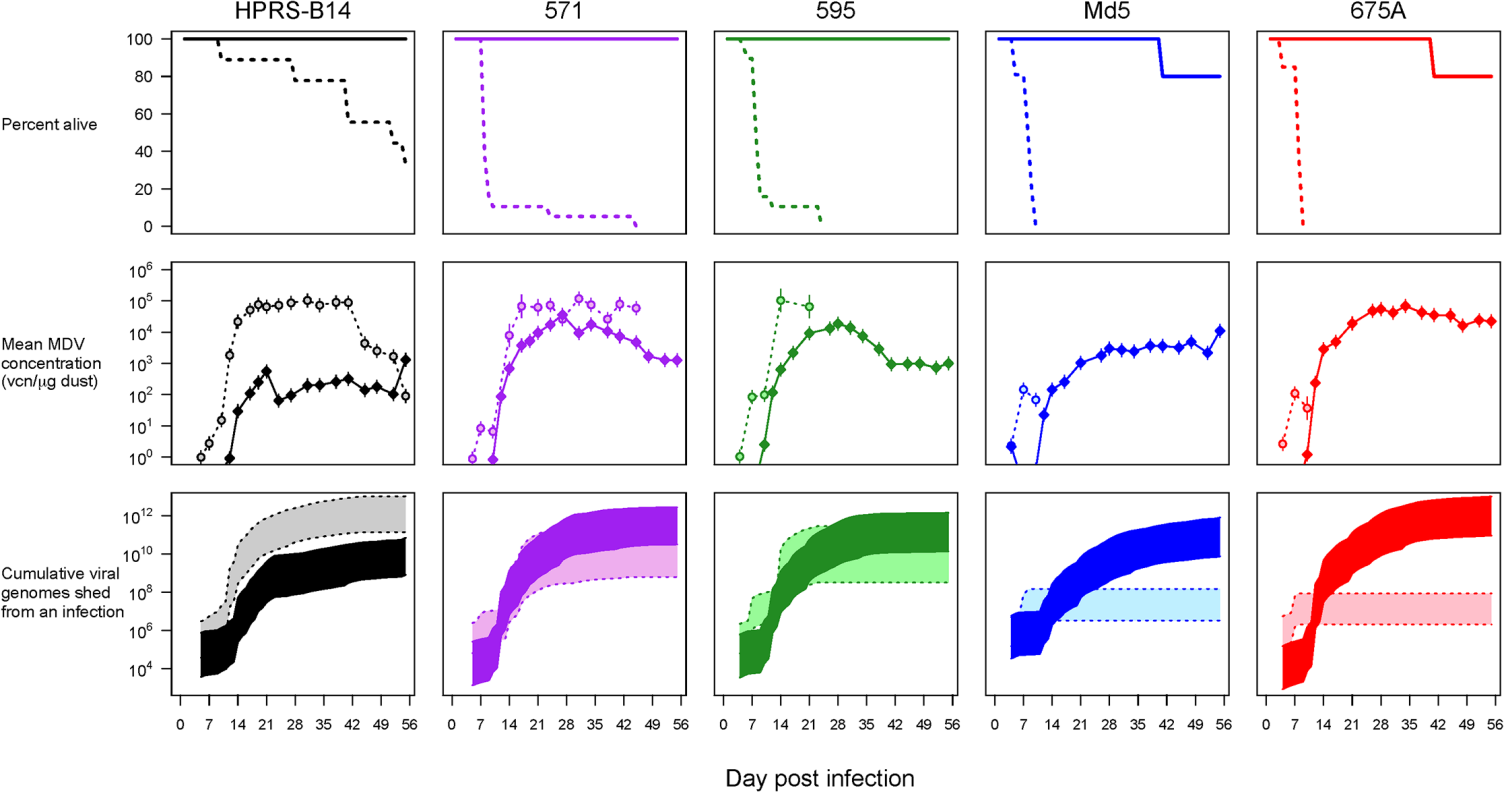
Impact of vaccination on mortality and viral shedding of five strains of MDV. Experiment 1. Groups of 20 Rhode Island Red chickens were unvaccinated (dotted lines, light shading) or HVT-vaccinated (solid lines, dark shading) at 1 d of age and challenged with viral strains HPRS-B14 (black), 571 (purple), 595 (green), Md5 (blue), or 675A (red) 8 d later. Viral strains vary in virulence in unvaccinated hosts, and vaccination protects against death (**top panels**, with strains arranged in order of increasing virulence from left to right.). Vaccination suppresses the concentration of virus in dust, but by keeping hosts alive, prolongs the infectious periods of hyperpathogenic MDV (**middle panels**). This means that cumulative number of virus genome copies (VCN) shed per bird is suppressed by vaccination for the least virulent strain and enhanced by several orders of magnitude for the most virulent (**bottom panels**). Error bars and shaded regions indicate 95% confidence interval (c.i.) Raw data can be found at http://dx.doi.org/10.5061/dryad.4tn48.

Our first three experiments involved Rhode Island Red (RIR) chickens, a breed that has not been subject to the intensive selective breeding and outcrossing that characterizes modern commercial chicken strains. Specific pathogen-free (SPF) parent birds were unvaccinated, and so offspring used in our first two experiments were free from maternally derived antibodies. In our first experiment, we infected 8-d-old chicks with five strains of MDV chosen to span the virulence spectrum defined by Witter and colleagues [21,29]. The viral strains varied from the less virulent HPRS-B14, which killed 60% of unvaccinated birds over 2 mo, to the highly lethal Md5 and 675A, which killed all unvaccinated birds in 10 d (Fig 1, top panels). When age-matched birds were vaccinated 8 d earlier with HVT, the first MDV vaccine to go into commercial use, survival improved dramatically, with a few deaths occurring only late in the experiment, and then only in birds infected with the most virulent strains (Fig 1, top panels).

We collected dust from the isolators containing infected birds and measured the concentration of virus genomes in the dust using real-time PCR. At contemporaneous time points, vaccinated birds shed fewer virus genome copies than unvaccinated birds infected with the same viral strain (Fig 1, middle panels). Those patterns reflected viral loads in the feather follicles (S1 Fig). Critically, the infectious period of unvaccinated birds infected with our two most virulent strains was less than a week because hosts died so rapidly. During that week, barely any virus was shed (Fig 1, middle panels). In contrast, the infectious period of the least virulent strains continued for the entire experiment (almost 2 mo). Thus, the least virulent strain shed several orders of magnitude more virus from unvaccinated birds than did the virulent strains (Fig 1, bottom panels). By preventing death, vaccination greatly increased the infectious period of the most virulent strains, increasing the total amount of virus shed by several orders of magnitude, and increasing it above that of the least virulent strain (Fig 1, bottom panels). Thus, the net effect of vaccination on both host survival rates and daily shedding rates was to vastly increase the amount of virus shed by virulent strains into the environment.

### Onward Transmission

**Fig 2:**
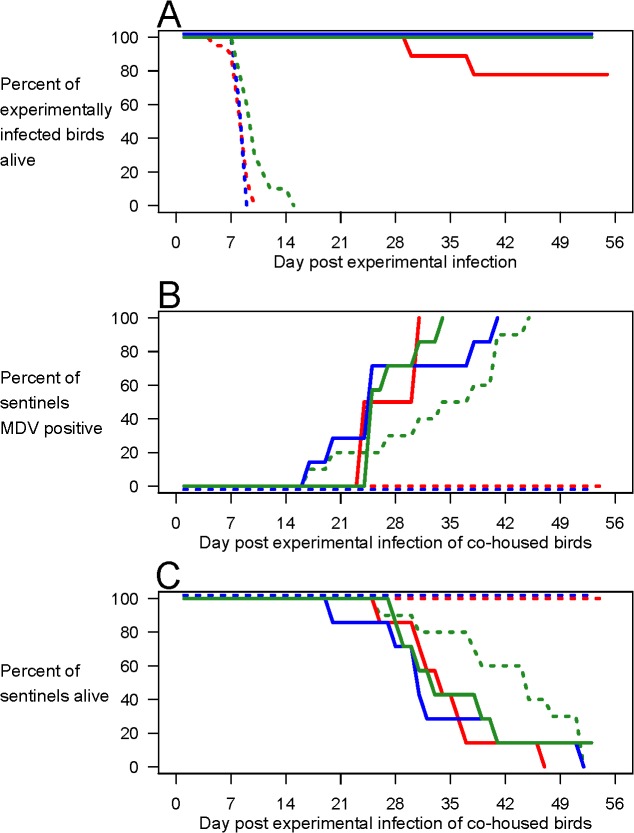
Vaccination enhances transmission of hyperpathogenic MDV. Experiment 2. Groups of ten birds were HVT-vaccinated (solid lines) or not (dotted lines) and experimentally infected with one of our three most virulent MDV strains, 595 (green), Md5 (blue) and 675A (red), and co-housed with ten unvaccinated sentinel birds. Vaccination prolonged the survival of experimentally infected birds (**A**), ensuring that sentinel birds became infected (**B**) and, hence, died (**C**). In B and C, solid lines denote sentinels cohoused with vaccinated experimentally infected birds and dotted lines denote sentinels cohoused with unvaccinated experimentally infected birds. Raw data can be found at http://dx.doi.org/10.5061/dryad.4tn48.

To confirm that virus shed into the environment was a robust proxy for overall bird-to-bird transmission potential, we co-housed birds infected with our three most virulent strains with immunologically-naïve sentinel birds (Experiment 2). When unvaccinated birds were infected with the two most lethal strains (Md5 and 675A), they were all dead within 10 days (Fig 2A), before substantial viral shedding had begun (S2 Fig). Consequently, no sentinel birds in those isolators became infected (Fig 2B) and none died (Fig 2C). In contrast, when HVT-vaccinated birds were infected with either of those hyperpathogenic strains, they survived for 30 days or more (Fig 2A), allowing substantial viral shedding (S2 Fig). All co-housed sentinels consequently became infected (Fig 2B) and went on to die as a result of MDV infection (Fig 2C). Thus, in accordance with the imperfect-vaccine hypothesis, vaccination enabled the onward transmission of viruses otherwise too lethal to transmit, putting unvaccinated individuals at great risk of severe disease and death.

Interestingly, the viral strain 595 was slightly less virulent than the other two viruses (taking a day longer to kill half of the unvaccinated birds, and 6 d longer to kill them all) (Fig 2A). This slightly reduced mortality rate prolonged the viral shedding from unvaccinated birds, so that about 100-fold more virus was shed into the environment by the 595-infected cohort than from the cohorts infected by the two more lethal strains (S2 Fig). This was evidently sufficient for transmission, because all co-housed sentinels eventually became infected (Fig 2B) and went on to die (Fig 2C). Thus, slight reductions in lethality can be sufficient to allow onward transmission. Nonetheless, even for strain 595, vaccination led to more rapid infection of sentinels (Fig 2B; median time to positivity 9 d earlier than in unvaccinated birds, *p* < 0.05), thus increasing the rate at which secondary cases were generated, a critical determinant of both viral fitness and case incidence in a rising epidemic.

### Maternally Derived Antibody

The high mortality rates we observed in unvaccinated chickens infected with our most virulent strains are due to early mortality syndrome, which involves the rapid onset of paralysis, disorientation and an inability to feed and move, followed by death [30–33]. In today’s modern industry, parental birds are almost always vaccinated against MDV, which results in the transfer of maternal antibody to chicks. These antibodies appear to be protective against the early mortality syndrome [30–33]. This raises the prospect that vaccination of laying hens could also permit onward transmission of viral strains that would be too lethal to otherwise transmit from offspring birds. We tested this possibility with further experiments using our most (675A) and least (HPRS-B14) virulent virus strains, again in Rhode Island Red birds, but this time including chicks derived from hens vaccinated 4–5 wk prior to egg lay with a standard commercial Rispens vaccine (Experiment 3).

**Fig 3:**
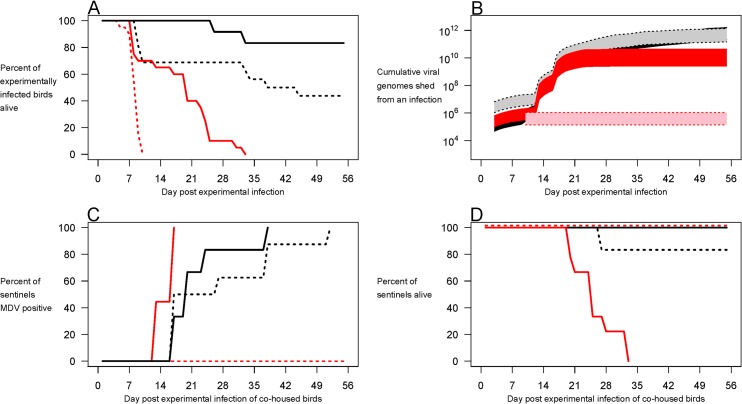
Maternal vaccination enhances viral shedding and onward transmission of hyperpathogenic MDV. Experiment 3. Groups of ten unvaccinated chicks produced by hens that were Rispens-vaccinated (solid lines) or not (dotted lines) were infected with viral strains HPRS-B14 (black) or 675A (red) and cohoused with sentinels of the same maternal antibody status. Maternally derived antibodies prolonged the survival of experimentally infected birds (**A**) and enhanced the amount of virus shed into the environment by the hyperpathogenic strain (**B**), making possible the infection of sentinels with the most virulent strain (**C**), which led to their death (**D**). Shaded regions represent 95% c.i. for unvaccinated (light) and vaccinated (dark). Raw data can be found at http://dx.doi.org/10.5061/dryad.4tn48.

Vaccination of hens enhanced the survival of offspring experimentally infected with HRPS-B14 (Fig 3A, *p* < 0.05). Maternally derived antibody had no detectable effect on the replication of that viral strain in the feather tips (S3 Fig panel A, *p* > 0.05) and, while it somewhat suppressed the amount of infectious virus shed into the environment early in infections (Figs 3B and S3B), it did not affect the rate at which sentinel birds became infected with HRPS-B14 (Fig 3C, *p* > 0.05) and few sentinels died (Fig 3D). Thus maternal protection had little impact on the transmission success of our least virulent strain.

However, presence of maternal antibody greatly impacted the transmission success of the most virulent strain (675A). As expected, the offspring of vaccinated hens survived for longer following infection with 675A virus than did maternally derived antibody-negative chicks (Fig 3A, *p* < 0.05). As we found in our first two experiments, very little of the highly virulent strain was shed from birds with no immune protection before they died (Figs 3B and S3). Consequently, no sentinels became infected (Fig 3C). But birds with maternal protection survived longer to shed more virus (Figs 3A, 3B, and S3B), so that all sentinel birds became infected (Fig 3C) and died (Fig 3D). Maternal vaccination was not as protective as direct vaccination of offspring (cf. Fig 3A with Fig 2A and the top panels of Fig 1). Nonetheless, vaccination of laying hens, like the vaccination of offspring, enabled the onward transmission of the hyperpathogenic strain from offspring (Fig 3C). Again, these data are consistent with the imperfect-vaccine hypothesis.

### Transmission between Commercial Birds

Our experiments above show that direct vaccination of birds or vaccination of parent hens makes possible the onward transmission of viral strains otherwise too lethal to transmit, and thus that unvaccinated individuals are put at increased risk of severe disease and death. However, in a modern commercial broiler setting, all birds in a flock would originate from vaccinated hens (and so would be positive for maternally derived antibody), and also be vaccinated. We thus set out to determine whether our most virulent strain could transmit to vaccinated sentinels, a necessary condition for persistence of hyperpathogenic strains in the modern industry (Experiment 4). To mimic the current commercial broiler situation, we obtained modern commercial broiler birds derived from Rispens-vaccinated hens and, at 1 d of age, we HVT-vaccinated all the birds we would experimentally infect. Those birds were then infected with our most virulent viral strain (675A) at 8 d of age. We cohoused those experimentally infected birds with sentinel birds, which were either HVT vaccinated or not. We performed this experiment twice. To accommodate changing regulatory requirements (see Methods), we did the first replicate with birds housed in isolators until 35 d of age, after which they were moved to floor until they were 11 wk old (Experiment 4a), and the second replicate with birds maintained in floor pens from 1 d of age until 7 wk of age (Experiment 4b).

**Fig 4:**
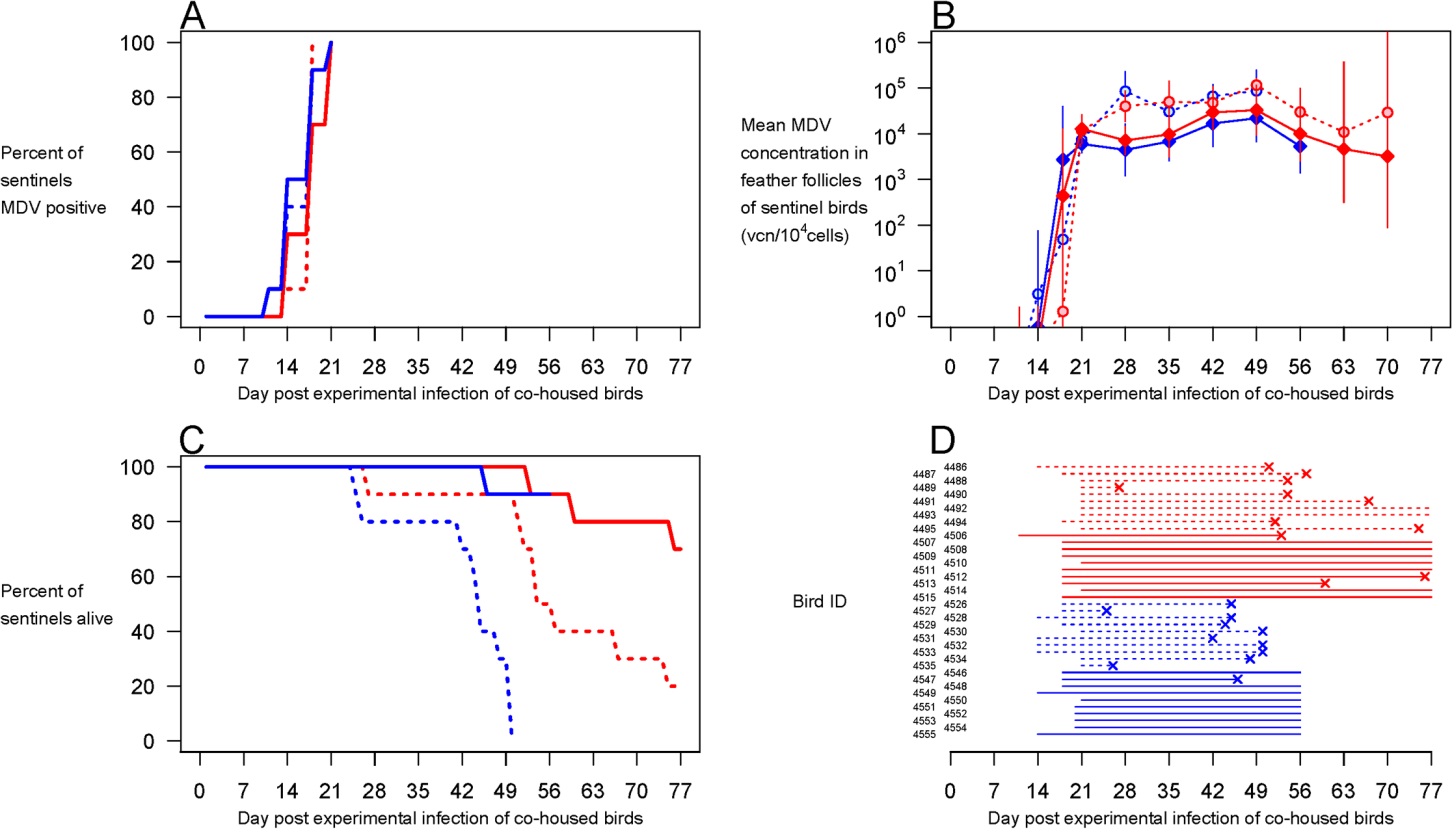
Transmission of hypervirulent MDV to modern commercial birds. Experiment 4. Groups of ten modern commercial broiler chicks derived from Rispens-vaccinated hens were HVT-vaccinated at 1 d of age and experimentally infected with the hypervirulent MDV strain 675A. Those experimentally infected birds were co-housed with groups of ten sentinel birds from the same commercial stock (and thus also derived from Rispens-vaccinated hens) which were HVT-vaccinated (solid lines) or not (dotted lines). The experiment was performed twice (experiment 4a, red; experiment 4b, blue). Independent of their vaccine status, all sentinel birds became infected (**A**), with high levels of virus replication in feather follicles (**B**). Vaccination prolonged the survival of sentinel birds (**C**), and consequently their infectious period (**D**). In panel D, “x” denotes death because of MDV. Error bars, 95% c.i. Raw data can be found at http://dx.doi.org/10.5061/dryad.4tn48.

All sentinels became infected, irrespective of vaccine status (Fig 4A). Thus, vaccinated maternal antibody positive commercial birds shed wild-type virus that caused infections in both vaccinated and unvaccinated maternal antibody positive birds. Vaccination only slightly suppressed viral replication in the infections acquired by the sentinel birds (Fig 4B). Importantly, all sentinels, vaccinated and unvaccinated, became virus positive in the feather follicles, meaning that they themselves started shedding. Vaccination protected sentinel birds from death (Fig 4C), prolonging infectious periods by about 2 wk (Fig 4D; standard error of the difference ±3.2 d, F_1,36_ = 19.9, *p* < 0.0001). Thus, not only does our most virulent strain transmit between modern commercial broilers when they are vaccinated, the duration of shedding in the next step in the transmission chain is also increased by vaccination.

## Discussion

MDV became increasingly virulent over the second half of the 20th century [19,21–24]. Until the 1950s, strains of MDV circulating on poultry farms caused a mildly paralytic disease, with lesions largely restricted to peripheral nervous tissue. Death was relatively rare. Today, hyperpathogenic strains are present worldwide. These strains induce lymphomas in a wide range of organs and mortality rates of up to 100% in unvaccinated birds. So far as we are aware, no one has been able to isolate non-lethal MDV strains from today’s commercial (vaccinated) poultry operations [19,23]. Quite what promoted this viral evolution is unclear. The observation that successively more efficacious vaccines have been overcome by successively more virulent viral strains has prompted many MDV specialists to suggest that vaccination might be a key driver [19–24,34–37], though identifying the evolutionary pressures involved has proved challenging. There is no evidence in Marek’s disease that vaccine breakthrough by more virulent strains has anything to do with overcoming strain-specific immunity (e.g., epitope evolution); genetic and immunological comparisons of strains varying in virulence suggest that candidate virulence determinants are associated with host–cell interactions and viral replication, not antigens [19]. The imperfect-vaccine hypothesis was suggested as an evolutionary mechanism by which immunization might drive MDV virulence evolution [2], but there has been no experimental confirmation. Our data provide that: by enhancing host survival but not preventing viral shedding, MDV vaccination of hens or offspring greatly prolongs the infectious periods of hyperpathogenic strains, and hence the amount of virus they shed into the environment.

Our data do not demonstrate that vaccination was responsible for the evolution of hyperpathogenic strains of MDV, and we may never know for sure why they evolved in the first place. Clearly, many potentially relevant ecological pressures on virulence have changed with the intensification of the poultry industry. For instance, as the industry has expanded, broilers have become a much larger part of the industry, and broiler lifespans have halved with advances in animal genetics and husbandry; all else being equal, this would favour more virulent strains [28], so too might greater genetic homogeneity in flocks [38] or high-density rearing conditions [13], or indeed increased frequencies of maternally derived antibody if natural MDV infections became more common as the industry intensified in the pre-vaccine era (Fig 3) [39]. But whatever was responsible for the evolution of more virulent strains in the first place (and there may be many causes), our data show that vaccination is sufficient to maintain hyperpathogenic strains in poultry flocks today. By keeping infected birds alive, vaccination substantially enhances the transmission success and hence spread of virus strains too lethal to persist in unvaccinated populations, which would therefore have been removed by natural selection in the pre-vaccine era.

The relaxation of natural selection against hyperpathogenic strains revealed by our experiments arises because vaccination enhances host survival. In serial passage experiments with a rodent malaria, immunity induced by whole parasite immunization [40] or vaccination with a recombinant antigen [10] also promoted the evolution of virulence. However, by design, those experiments did not allow host death to impact pathogen fitness, and so the evolution towards increased virulence was driven in a different way. Evidently, immunity in that system is disproportionately efficacious against less virulent strains. Our MDV experiments were not designed to test for within-host selection, but there is some suggestion that vaccine-induced immunity better controlled the replication of the least virulent strain (Figs 1 and S1). In principle, these two evolutionary pressures (within-host selection favouring virulent variants for their ability to evade immunity and vaccine-induced relaxation of between-host selection against virulence) could together generate very potent selection for more virulent strains [4]. Within-host competition between strains could add further selection for higher virulence [41,42].

Vaccine failure in the face of virulent pathogens has been documented for at least two viruses other than MDV: feline calicivirus [43] and infectious bursal disease virus in poultry [44]. Both cases are also associated with long-term use of leaky anti-disease vaccines. Our data are also consistent with hypotheses purporting to explain virulence increases in two well-studied wildlife systems. First, strains of the poultry pathogen *Mycoplasma gallisepticum* in North American house finches have become increasingly virulent, probably due to the increasing incidence of partially immune birds after the bacterium emerged in finch populations in the 1990s [45]. Second, after well-documented declines in virulence following its release as a biocontrol agent in Australia, myxoma virus became increasingly virulent; that virulence evolution was most likely a consequence of increases in the genetic resistance and hence survival of wild rabbits in response to natural selection imposed by the virus [46]. In both cases, anti-disease protection induced by natural immunization (finches) or by genetic resistance (rabbits) prolonged the infectious periods of otherwise highly lethal strains. These cases and our data raise the prospect that a variety of disease mitigation technologies have the potential to drive virulence evolution, including disease-ameliorating drugs [7,47] or genetic enhancements of host resistance [48]. If these technologies prolong infectious periods of hyperpathogenic strains, as we have shown vaccination can, they too could create conditions favouring the emergence of highly lethal strains. This does not mean that such technologies should be avoided, particularly when alternative options are limited. Vaccination has massively reduced yield losses due to MD, despite the evolution [49]. However, when protecting all individuals is impossible, or evolution is ongoing, the use of additional transmission-blocking interventions such as improved hygiene might be essential.

We suggest that the risk of outbreaks of hyperpathogenic strains be considered wherever disease interventions improve host survival without preventing pathogen transmission. Such situations might include vaccination against Newcastle disease [50] and avian influenza in poultry [51–53] and vaccination against *Brucella* in domesticated mammals [54], as well as genetic enhancement of agricultural animals including fish and poultry. Whether leaky human vaccines could also create the conditions in which more virulent strains can thrive will depend, among other things, on the selective factors currently preventing the emergence of hyperpathogenic strains in human populations. Our data emphasize that a comprehensive understanding of the impact of vaccines on pathogens cannot end with Phase III clinical trials or post-implementation studies of antigenic or serotype frequencies [2,4,10,55].

## Materials and Methods

### Vaccine and Challenge Virus Strains

The HVT vaccine virus strain FC126 was second chick embryo fibroblast (CEF) passage stock from commercial HVT vaccine (Poulvac, Fort Dodge Animal Health). Commercial CVI988/Rispens vaccine virus (Nobilis Rismavac) was from Intervet. The challenge virus strains (seventh duck embryo fibroblast passage stocks) were a gift from Dr. A. M. Fadly (Avian Disease and Oncology Laboratory, United States). In the MDV literature, virulence (pathotype) is defined in terms of vaccine break-through [21,27,29], with virus strains categorized into pathotypes denoted as mild, virulent, very virulent, or very virulent plus (mMDV, vMDV, vvMDV, vv+MDV). In our experiments, we used up to five strains chosen to cover this spectrum. The strains were HPRS-B14, 571, 595, Md5, and 675A. HPRS-B14 has not been formally pathotyped, but would likely be categorised at the lower end of vMDV. The remaining four of these strains have been pathotyped as vMDV, vvMDV, vvMDV, and vv+MDV respectively [21]. Note, however, that for the purposes of the present paper, defining virulence in terms of vaccine resistance introduces semantic circularity. Consequently, in the main text and what follows here, we instead explicitly define (measure) virulence as lethality in immunologically naïve birds.

For amplification of virus stocks, and to ensure there was no variation in virus passage history between experiments, 5-d-old Rhode Island Red (RIR) chickens were inoculated with 1,000 plaque forming units (pfu) of virus, via the intra-abdominal route. Lymphocytes isolated from spleens harvested at 14 d post infection (dpi) were cultured with primary CEF cells for 7 d, when cytopathic effect was clearly visible. The cells were passed two further times in CEF to produce virus stocks. The cell-associated virus stocks were titrated and stored in liquid nitrogen. CVI988/Rispens and HVT vaccines were administered via the subcutaneous route (neck), and challenge virus via the intra-abdominal route.

### Experimental Chickens and Experimental Design

All studies and procedures involving animals were in strict accordance with the European and United Kingdom Home Office regulations and the Animals (Scientific Procedures) Act 1986 Amendment Regulations 2012, under the authority of the Project Licenses PPL 30/2621 and PPL 30/3169. Birds were individually identifiable with wing bands and had access to water and a vegetable-based feed ad libitum. Any bird deemed to have reached the humane endpoint was culled. In the main text, the humane endpoint was taken as the time at which “infection-induced death” occurred. Chickens which reached the humane endpoint from 5–10 dpi (early mortality phase) showed a rapid onset of paralysis, disorientation, reluctance to feed, reluctance to move. and reduced weight gain. In our experience, this endpoint precedes natural death by less than two hours. Chickens which reached the humane endpoint from 15 dpi onwards showed a gradual onset of reluctance to feed, lethargy, and reduced weight gain. In our experience, this endpoint precedes death by up to 24 h. These endpoints, and our estimates of their timing with respect to viral-induced death, were arrived at from small-scale pilot experiments that determined the necessity for close monitoring because of the rapid onset of virus-induced death. Any bird that was found dead was reported to the UK Home Office. The majority of culled chickens showed enlarged spleen with gross lymphoid lesions. The prevalence of visceral lesions was broadly in line with those described by Witter (1997, his Table 4) for strains of corresponding pathotypes, despite differences in the breed and maternal antibody status of test chickens, and slight differences in the passage number of viral stocks.

For Experiments 1–3, chickens of the outbred Rhode Island Red (RIR) breed were hatched from the eggs of specified-pathogen-free flocks maintained at The Pirbright Institute. Chicks hatched from the eggs of unvaccinated hens were considered free from maternally derived antibody against MDV, and are hereafter referred to as MtAb-neg chicks. Chicks having maternally derived antibody against MDV (MtAb-pos) were hatched from eggs collected from RIR hens 4–5 wk after these hens were vaccinated with one commercial dose of Nobilis Rismavac CVI988/Rispens MDV vaccine (Intervet).

In Experiments 1–3, chicks were housed in positive pressure, high efficiency particulate air (HEPA)-filtered avian isolators (Controlled isolation Systems, US) within rooms in the Experimental Animal House at The Pirbright Institute, Compton. Chickens were monitored up to four times daily, and any chicken considered to have reached the humane endpoint was culled by cervical dislocation. When experiments were terminated, any surviving birds were culled. Post mortem examination was performed on all culled chickens and the presence or absence of gross Marek’s disease lesions recorded.

The isolators are designed to house 20 1-d-old chickens or five adult chickens. In groups in which mortality following infection was low, it was necessary to reduce crowding in an isolator at intervals, by culling some birds. In these cases, birds to be culled were randomly selected, and the number of infected and sentinel birds culled was arranged to maintain the appropriate infected:sentinel ratio. Any birds culled for the purposes of reducing crowding were not included in survival data calculations.

For experiments 4a and b, commercial broiler breed chicks of the “Aviagen slow growing broiler line” were hatched from eggs supplied by Aviagen. Eggs were from CVI988/Rispens vaccinated hens, and therefore, all birds used were MtAb-pos as confirmed by ELISA (see below). We used different housing protocols for Experiment 4 from those adopted in Experiments 1–3 because regulatory requirements changed over the course of our studies, with work with adult birds in isolators becoming strongly discouraged. This meant we moved to floor-housing birds for at least part of Experiment 4, a condition that, anyway, more closely resembles housing conditions in the poultry industry. In Experiment 4a, birds were housed in isolators (as described above) until they were 35 d of age when groups were moved into floor pens within separate experimental rooms. Floor pens were constructed from metal barred caging panels that could have sections added to the layout to increase the pen area as birds became larger. Compressed straw pellets were used as bedding. In Experiment 4b, birds were housed within floor pens from 1 d of age in separate experimental rooms. To restrict the dissipation of dust and dander in first few weeks, the initial floor pen (measuring 1 m × 1 m) was partially contained using Perspex sheets attached to the wire caging to form a housing cube with open edges. As birds increased in size, additional non-Perspex covered cage sections were added to the initial cube to increase the pen area.

### Experiment 1: Effect of HVT-Vaccination on Shedding of Five Strains of MDV

Two hundred (*n* = 200) MtAb-neg 1-d-old chicks were randomly allocated to ten groups, each group of 20 chicks being housed in a separate isolator, with two isolators (A and B) per room (S1 Table). In each room, chicks in one isolator (A) were vaccinated with HVT at 1 d of age, while chicks in the second isolator (B) were not vaccinated. At 8 d post vaccination (dpv), all chicks were challenged with one of five strains of MDV, each strain being used to infect a group of unvaccinated chicks, and a group of HVT-vaccinated chicks (S1 Table). Doses of vaccine and challenge viruses were approximately 1000 pfu and 300–600 pfu per chicken, respectively.

From each group, ten pre-selected chicks were feather-sampled [56,57] twice weekly until 55 dpi or until they reached the humane endpoint. Dust samples were also collected from each isolator twice weekly or until no chickens remained. Each time dust samples were taken, the pre-filter on the isolator air exhaust was removed and replaced with a new, clean filter. Within the isolator, the removed filters were shaken into a polythene bag to collect poultry “dust,” which was transferred to tubes and stored at −20°C.

### Experiment 2: Effect of HVT-Vaccination on Transmission of Three Strains of MDV

One hundred and twenty (*n* = 120) MtAb-neg 1-d-old chicks were randomly allocated to six groups, each group of 20 chicks being housed in a separate isolator, and there being two isolators (A and B) per room (S2 Table). In each group, ten chickens were randomly selected to be the “shedder” birds (i.e., experimentally infected), while the remaining ten chickens were selected to be “in-contact sentinel birds”. In one isolator from each room (A isolators), the shedder birds were vaccinated with HVT at one day of age. In B isolators, the shedder birds were not vaccinated. At 8 dpv all shedder birds were challenged with one of three strains of MDV. Sentinels were neither vaccinated nor challenged. Doses of vaccine and challenge viruses were approximately 2,750 pfu and 1,000–1,500 pfu per chicken, respectively.

Sentinel birds are necessary to directly measure natural transmission rates, but once sentinels themselves become infectious, they make it difficult to determine how much virus is being shed by experimentally infected birds. For our studies of 675A, we therefore added two additional treatment groups (Groups 4A and 4B, S2 Table) by randomly allocating 20 (*n* = 20) additional MtAb-neg 1-d-old chicks to two additional isolators, with ten birds in one isolator being vaccinated as above (A isolator), and the ten in the other isolator not being vaccinated (B isolator). All 20 of those additional birds were then experimentally infected at 8 dpv, as above. The existence of these two extra groups allowed us to estimate the viral shedding rates for birds experimentally infected with 675A without any issue of viral contamination from sentinels. We did not, however, have the resources to run analogous extra groups for Md5 or 595, and so the dust data for those strains (S2 Fig) includes dust shed from sentinel birds, which may contain virus after about 20 d post-experimental infection, when the first sentinels began to become infectious (Fig 2, S2 Fig).

Feather samples were collected from every shedder bird cohoused with sentinels twice weekly until 52 dpi or until they reached the humane endpoint. From 3 d onwards, at the same time points unless there were none alive, 150 μL blood samples were collected from every sentinel into 3% sodium citrate. At the same times, dust was collected and stored as described above. For the analysis of the 675A data, we used the survival data for all 20 experimentally infected birds of identical vaccine status (i.e., pooling the relevant data from the Group 1 and Group 4 isolators), the dust data (S2 Fig panel B) from the Group 4 isolators and the feather data (S2 Fig panel A) from the Group 1 isolators (groups defined in S2 Table).

### Experiment 3: Effect of Maternally Derived Antibody on Shedding and Transmission of Two Strains of MDV

Sixty (*n* = 60) MtAb-neg 1-d-old chicks and 60 MtAb-pos 1-d-old chicks were randomly allocated to groups, each group being housed in a separate isolator, and there being two isolators (A, containing 20 chicks, and B, containing ten chicks) per room (S3 Table). In A isolators, ten chickens were randomly selected to be shedder birds (i.e., experimentally infected), while the remaining ten chickens were selected to be in-contact sentinels. At 9 d of age, all shedder birds were challenged with one of two strains of MDV; sentinels were not challenged. In each B isolator, all ten chickens were challenged with one of two strains of MDV, and these isolators were used for collection of dust so that shedding of MDV from ten infected chickens could be accurately determined without “dilution” by dust from non-infected sentinels. Doses of challenge viruses were 100–500 pfu per chicken.

Blood (150 μL) was collected from five pre-selected chickens from each of the B isolators prior to challenge. Serum was stored at −20°C. Feather samples were collected from every shedder bird in the A isolators twice weekly until 52 dpi or until they reached the humane endpoint. From 3 dpi onwards, blood samples were collected from every sentinel bird at these same time-points (or until such time that no chickens remained). At each of the above time-points (or until such time that no chickens remained), dust was collected from B isolators and stored as described above.

All tested chickens from the MtAb-neg group were negative for anti-MDV antibody (assayed by ELISA; see below), while all tested chickens from the MtAb-pos group were positive for maternal antibody.

### Experiments 4a and b: Transmission between Commercial Birds

In both Experiments 4a and 4b, 40 (*n* = 40) commercial broiler breed chicks were hatched from eggs produced by CVI988/Rispens vaccinated hens and therefore all were MtAb positive. The basic experimental design for Experiments 4a and b was the same; four test groups of age-matched chicks; two groups acting as MtAb-pos, HVT vaccinated, experimentally infected shedder birds housed independently with one of two groups of MtAb-pos sentinel birds that differed in their vaccination status, i.e., either HVT vaccinated or not (see S4 Table).

In both Experiments 4a and 4b, 40 1-d-old chicks were randomly placed into four groups across four isolators (S4 Table). In each experiment, all birds except the ten within each unvaccinated sentinel group were vaccinated with approximately 1,500 pfu HVT FC126 via the subcutaneous route at one day of age. At 8 d of age, the two groups serving as shedders were challenged with approximately 725 pfu of vv+ MDV 675A via the intra-abdominal route. Blood (150 μL) and feather samples were collected twice weekly from all birds until 21 dpi and thereafter weekly. Dust samples were collected weekly from the housing air extract filters, as above.

### Preparation of DNA and Real-Time PCR for PBL, Feather, and Dust Samples

Viral titres were assayed indirectly by PCR as follows. Peripheral blood lymphocytes (PBL) and feather tip samples were prepared as previously described [56,57]. Each dust sample was measured into triplicate 5 mg aliquots. Total DNA was prepared from each PBL, feather and dust sample using a DNeasy-96 kit (Qiagen), according to the manufacturers’ instructions for extraction of DNA from cells (PBL) or from tissues (feather tips and dust). Real-time quantitative duplex PCR (q-PCR) to amplify the MDV-1 *meq* gene and the chicken *ovotransferrin* (*ovo*) reference gene was used for absolute quantification of MDV genomes as previously described [56]. This assay does not detect HVT vaccine virus. All reactions using feather tips or dust samples as target DNA contained 10 μg bovine serum albumin to overcome the inhibitory effect of melanin pigment [56]. Standard curves, prepared using 10-fold serial dilutions of DNA from MDV1-infected CEF (for *meq* reaction) and non-infected CEF (for *ovo* reaction) and accurately calibrated against plasmid constructs of known target gene copy number, were used to quantify MDV genomes per 10^4^ cells or per μg dust.

### Measurement of Maternally-Derived Antibody against MDV

Enzyme-linked immunosorbent assay (ELISA) was performed to measure maternally derived antibody against MDV in serum samples from hatched chicks [58]. Serum samples were tested in duplicate at 1:100 dilution on ELISA plates coated with MDV-infected or non-infected cell lysates. Serum from a non-infected chicken was used as a negative-control, and serum from an MDV1-infected chicken as a positive control.

### Statistical Analyses

All data and the R code used to create all the figures are deposited in the Dryad repository: http://dx.doi.org/10.5061/dryad.4tn48 [59].

For groups of chickens, mean values for virus genome copy number for PBL, feather tips or dust, were determined using the log_10_ transformed copy number for each individual sample. For feather tip data, 95% c.i. of the means were calculated using the t-distribution. For dust data, 95% c.i. of the mean data were approximated as ±2 standard errors. Plotted values of virus concentration in dust are for samples based on the cumulative dust shed since the previous plotted sample (when filters were last changed). For each sentinel chicken, the time at which MDV was first detected in PBL by qPCR (time to positivity) was recorded. Time to positivity was taken as the first sampling time at which the q-PCR Meq Ct value was <40 for successive time-points. Statistical comparisons of survival or time to positivity were made using the Mantel-Cox test applied to Kaplan-Meier survival curves plotted using GraphPad Prism v5.

To assess transmission potential, a key component of viral fitness, we calculated the cumulative virus genome copy number (VCN) shed rate over the lifetime of an infection. Cumulative VCN shed is a good proxy for transmission potential because virus shed from feather follicles is the only source of infectious MDV, and because MDV-contaminated dust remains infectious for many months [16–18]. Cumulative VCN shed can be uniquely determined from three components; the dust shed from a bird over time, the concentration of VCN per unit shed dust over time, and the lifespan of an infection. We directly measured the latter two values in Experiments 1–3, and the former value has been previously estimated [28]. Details of how we used these measures to calculate cumulative VCN are given in S1 Protocol.

In Experiment 4, we estimated viral genome concentration in feather follicles of sentinel birds. Because these birds were co-housed with experimentally infected birds, virus-negative feather shafts can become contaminated with dust from infected birds. We therefore set quantitative thresholds for virus positivity in feather pulp, as described in S2 Protocol. Duration of infectious period (Fig 4D) was taken as the time from when viral titres first exceeded this threshold until bird death.

## Supporting Information

S1 Fig Vaccination suppresses but prolongs viral replication in the feather tips of experimentally infected birds. Experiment 1. Groups of 20 Rhode Island Red chickens that were unvaccinated (dotted lines) or HVT-vaccinated (solid lines) at 1 d of age and challenged with viral strains HPRS-B14 (black), 571 (purple), 595 (green), Md5 (blue), or 675A (red) 8 d later. Virus genome copy numbers were estimated by qPCR from the pulp of feathers plucked from individual birds. Error bars are 95% c.i. of the mean. Large error bars are from time points where few birds remained alive. Raw data can be found at http://dx.doi.org/10.5061/dryad.4tn48.
(EPS)

S2 Fig Vaccination suppresses but prolongs viral replication in the feather tips of experimentally infected birds, increasing total viral genomes shed into the environment. Experiment 2. Groups of 20 Rhode Island Red chickens were unvaccinated (dotted lines, light shading) or HVT-vaccinated (solid lines, dark shading) at 1 d of age and challenged with one of our three most virulent viral strains 595 (green), Md5 (blue), or 675A (red) 8 d later. **Top panel** shows virus replication in the feather follicles, **middle panel** shows virus concentration in dust collected from isolator filters, and **lower panel** shows estimates of cumulative viral genomes shed from an experimentally infected bird. Error bars and shaded areas are 95% c.i. of the mean. Note that estimates of cumulative viral genomes shed from vaccinated 595- and Md5-infected birds are biased upwards after around day 20, when sentinels began to shed virus (see Methods and S2 Protocol for discussion). Raw data can be found at http://dx.doi.org/10.5061/dryad.4tn48.
(EPS)

S3 Fig Maternal vaccination prolongs the replication of the most virulent strain of MDV in feather tips of infected chicks and hence shedding. Experiment 3. Groups of ten unvaccinated chicks produced by hens that were Rispens-vaccinated (solid lines) or not (dotted lines) were infected with viral strains HPRS-B14 (black) or 675A (red). Viral genome concentration in feather follicles (**top panel**) and in dust (**bottom panel**). Error bars are 95% c.i. of the mean. Large error bars in top panel are from time points where only two birds remained alive; after day 41, only one unvaccinated HPRS-B14-infected bird remained alive and so there are no error bars. Raw data can be found at http://dx.doi.org/10.5061/dryad.4tn48.
(EPS)

S1 Protocol Calculation of cumulative virus genome copy number of lifetime of an infection (Fig 1, lower panels, and Figs 3B and S2) [28,60,61]. (DOCX)

S2 Protocol Controlling for background viral contamination of feather pulp (Experiment 4, Fig 4B and 4D). (DOCX)

S1 Table Design of Experiment 1: Effect of HVT-vaccination on shedding of five strains of MDV. (DOCX)

S2 Table Design of Experiment 2: Effect of HVT-vaccination on transmission of three strains of MDV. (DOCX)

S3 Table Design of Experiment 3: Effect of maternally-derived antibody on shedding and transmission of two strains of MDV. (DOCX)

S4 Table Design of Experiments 4a and 4b: Transmission of MDV strain 675A in commercial maternal-antibody–positive HVT-vaccinated birds. (DOCX)

## Abbreviations

c.i.: confidence interval
MD: Marek’s disease
MDV: Marek’s disease virus
RIR: Rhode Island Red
SPF: Specific pathogen-free
VCN: virus genome copy number
