# Supplementary material for: Imperfect Vaccination Can Enhance the Transmission of Highly Virulent Pathogens
Source: PLoS Biol. 2015 Jul 27;13(7):e1002198. doi: 10.1371/journal.pbio.1002198 (PMC4516275; doi:10.1371/journal.pbio.1002198)
Supplement: S1 Protocol — (DOCX) [file pbio.1002198.s004.docx]

**S1 Protocol. Calculation of cumulative virus genome copy number of lifetime of an infection (Fig. 1 lower panels, Fig. 3b, S2 Fig.).**

The total VCN shed on a particular day is given by the equation

V_i_ = (1-p_i_) D_i_ Q_i_.

Here, V_i_ is the VCN shed on day i, p_i_ is the fraction of focal birds that have died from MDV infection by day i, D_i_ is the total dust shed by a bird on day i, and Q_i_ is the virus copy number per unit dust on day i. The cumulative VCN shed C_i_ is then given by

C_i_ = Σ_0_^i^ (V_i_).

In practice, we measured VCN concentration in shed dust, Q_i_, in up to 18 of the 55 days that our experiments ran. VCN concentration on non-measured days was assumed equal to the VCN concentration of the first following measured day. Assuming instead that VCN concentration on non-measured days was equal to the previously measured concentration had no qualitative effect on the results. In the treatments where no final measurement was available on day 55 post-infection, this value was set equal to the final VCN concentration measured. Using instead the maximum VCN shed rate observed from all treatments again had no qualitative effect on the results.

VCN concentration in dust Q_i_ was measured in triplicate when sufficient dust was available (see Methods). An estimate of the measurement error in Q_i_ between technical replicates is therefore available at each time point when multiple measurements were taken. We estimated the sample standard deviation on the natural log of the data. To correct for small sample sizes, we scaled the sample standard deviation by the appropriate small sample size correction factor [1]. Examination of the relationship between the log mean and the estimate of the standard error revealed only a slight effect of the mean on the standard deviation. We therefore estimated global mean measurement errors for Experiments 1, 2 and 3 by averaging each of these unbiased sample standard deviations for each experiment to get values of 0.40, 0.36, and 0.37 respectively. These point estimates were used to calculate standard errors and, in turn, 95% confidence intervals approximated by ±2 standard errors.

For experiments 1 and 3, where we were trying to estimate the reproducibility of the experiment, we considered two sources of biological variability: (1) bird to bird variation in VCN/µg dust and (2) bird to bird variation in survival. We accounted for the biological variation in VCN concentration Q_i_ shed from individual birds as follows. For welfare reasons (enforced by the UK Home Office), birds cannot be reared individually, and so rather than measure variation in VCN concentration in dust shed from individual birds, we instead measured VCN per 10^4^ host cells in bird feather tips for individual birds (S1-3 Figs.). This value is useful because it correlates closely with VCN concentration in dust [2]. We therefore assumed that biological variation in VCN concentration shed from individual birds was equal to the variation measured in VCN per 10^4^ host cells in feather tips. VCN per 10^4^ host cells was measured for up to ten birds on up to 18 days for each treatment. To calculate the variation between birds, we again natural log transformed these data before calculating the sample standard error. These estimates were again scaled by the small sample size correction factor relating to the number of birds tested, to provide unbiased estimates of the variation between birds, 133 estimates for Experiment 1 and 33 for Experiment 3. Using these data, we estimated that the standard deviation between birds due to biological variation was 2.17 in Experiment 1 and 0.97 in Experiment 3. The difference between these estimates may have been due to treatment-specific effects on virus shed variation, but using treatment-specific estimates of the standard deviation yielded similar results. We thus used a single estimate of the standard deviation for each experiment because we had no *a priori* reason to expect treatment to affect the variation in virus shedding.

As shown in the equations above, a key component of the cumulative VCN shed is the cumulative mortality of virus-infected birds over time (p_i_). Assessing mortality is very reliable in practice, meaning that technical variation is negligible. To account for biological variation in p_i_, we bootstrapped from our data by randomly sampling with replacement sets of birds equal to the focal number used in each treatment. Bird survival differed between the virus strains used in this experiment, and so survival curves were bootstrapped using birds only within treatments. This provided us with estimates of variation in survival that we might expect to see if the experiment were replicated.

The final component in the above equations is the dust shed from a bird over time D_i_. An equation as a function of bird age has been previously published [3]. In our analysis, we used the previously published equation with a finishing day (bird age) of 64, because this was the finishing day used in Experiments 1 and 3. Dust shed by birds is highly consistent and repeatable between experiments, and so we thought it unnecessary to include variation in this term. Moreover, changing the dust shed curve to other plausible curves had negligible effects on the cumulative dust shed curve.

Using these estimates of biological and technical variation, we then simulated 10,000 replicates of our experiment, and we calculated the cumulative VCN shed C_i_ from an average bird in each of these simulated experiments. The cumulative VCN through time is given as 95% c.i. (shaded regions in the lower panels in Fig. 1 and in Fig. 3b). For each treatment, we would expect 95% of replicate experiments to estimate that the cumulative VCN of an average bird falls in this interval.

For experiment 2, we were trying to estimate the error in the experimental measure of total virus shed by experimentally infected birds in that particular experiment (rather than likely experimental reproducibility), and so cumulative VCN for that experiment was calculated as above but with two differences: 1) we used only the observed survival and VCN/µg dust with measurement “technical” variation and did not include biological variation in these values, and 2) we used a correction on the measured VCN/µg dust (virus concentration * total birds alive / experimental birds alive) to account for the fact that these measured values include dust shed by sentinels. Thus the 95% c.i. in Fig. S2C represent the estimate for the mean VCN shed from an experimental bird in that experiment. Note that our corrected estimates of the VCN/µg dust from experimentally infected birds were calculated assuming that any sentinels present shed only un-infectious dust. This means that when infectious sentinels were present, our estimates will be biased upwards. This bias affects the data from only two of our six experimental groups – vaccinated birds infected with strains 595 and Md5 – and even then, only after about day 20 post experimental infection when the first sentinels began to shed virus (Fig. 2, S2 Fig.). The data for 675A-infected birds is not affected because we estimated viral shedding in isolators without sentinels for those experimental groups (S2 Table). The data for all three unvaccinated treatment groups are also unaffected because all experimental birds died before sentinels became infectious (Fig. 2, S2 Fig.). Note too that our conclusions in the main text are not dependent on quantitative estimates of viral shedding rates after day 20.

1. Bolch BW (1968) More on unbiased estimation of the standard deviation. Am Statistician 22: 27.

2. Baigent SJ, Kgosana LB, Gamawa AA, Smith LP, Read AF, Nair VK (2013) Relationship between levels of

very virulent MDV in poultry dust and in feather tips from vaccinated chickens. Avian Dis 57:440‐447.

3. Atkins KE, Read AF, Savill NJ, Renz KG, Islam A, Walkden‐Brown SW, et al. (2013) Vaccination and

reduced cohort duration can drive virulence evolution: Marek's diease virus and industrialized

agriculture. Evolution 67: 851‐860.
