# Supplementary material for: Imperfect Vaccination Can Enhance the Transmission of Highly Virulent Pathogens
Source: PLoS Biol. 2015 Jul 27;13(7):e1002198. doi: 10.1371/journal.pbio.1002198 (PMC4516275; doi:10.1371/journal.pbio.1002198)
Supplement: S2 Protocol — (DOCX) [file pbio.1002198.s005.docx]

**S2 Protocol. Controlling for background viral contamination of feather pulp (Experiment 4, Fig.s 4B, 4D)**

In Experiment 4, we estimated viral genome concentration in feather follicles of sentinel birds. Because these birds were co-housed with experimentally infected birds, virus-negative feather shafts can become contaminated with dust from infected birds. To control for this background noise, we considered viral replication to be occurring in feather pulp once there were more than 350 viral genomes/10^4^ host cells present. Concentrations below this level were found in samples from sentinel birds less than a week after experimentally-infected birds began shedding, which is earlier than sentinel birds could have become positive in the feather follicles and must therefore have been due to contamination. We therefore assumed each sample with <350 viral genomes/10^4^ host cells had zero virus when producing Fig. 4B & 4D, and then estimated for each of the 40 sentinel birds, the duration of infectiousness as the time from first positivity above the 350 viral genomes/10^4^ host cells threshold until bird death due to MDV or experimental euthanasia (Fig. 4B). We tested whether the vaccination affected this duration of infectiousness using a GLM fitting *vaccine status* (HVT or not), *experiment* (4a or 4b), and *vaccine*experiment* interaction. The conclusion that vaccination prolongs the shedding period of sentinel birds is unaltered if the ‘contamination’ threshold is raised from 350 to 1000 viral genomes/10^4^ host cells (shedding period prolonged by 15 days; s.e. of the difference ±3.25 days, F_1,36_=21.6, P<0.0001) or if we ignore the issue (shedding prolonged by 13 days ±3.13 days, F_1,36_=19.2, P<0.0001). The duration of infectiousness differed between experiments 4a and b because the experiments were terminated at different times post-infection, but the impact of vaccination did not differ between the experiments (*vaccine*experiment* interaction, P>0.55 for 0, 350 or 1000 viral genomes/10^4^ host cells threshold).
