# Supplementary material for: Imperfect Vaccination Can Enhance the Transmission of Highly Virulent Pathogens
Source: PLoS Biol. 2015 Jul 27;13(7):e1002198. doi: 10.1371/journal.pbio.1002198 (PMC4516275; doi:10.1371/journal.pbio.1002198)
Supplement: S1 Table — (DOCX) [file pbio.1002198.s006.docx]

**Table S1. Design of Experiment 1: Effect of HVT-vaccination on shedding of five strains of MDV**

| **Challenge virus** | **Room** | **HVT Fc126-vaccinated** | **Unvaccinated** |
| --- | --- | --- | --- |
| 675A (vv+MDV) | 1 | Group 1A  20 infected chicks | Group 1B  20 infected chicks |
| 595 (vvMDV) | 2 | Group 2A  20 infected chicks | Group 2B  20 infected chicks |
| Md5 (vvMDV) | 3 | Group 3A  20 infected chicks | Group 3B  20 infected chicks |
| 571 (vMDV) | 4 | Group 4A  20 infected chicks | Group 4B  20 infected chicks |
| HPRS-B14 (vMDV) | 5 | Group 5A  20 infected chicks | Group 5B  20 infected chicks |

Each of the ten groups was housed in a separate isolator
