# Supplementary material for: Imperfect Vaccination Can Enhance the Transmission of Highly Virulent Pathogens
Source: PLoS Biol. 2015 Jul 27;13(7):e1002198. doi: 10.1371/journal.pbio.1002198 (PMC4516275; doi:10.1371/journal.pbio.1002198)
Supplement: S2 Table — (DOCX) [file pbio.1002198.s007.docx]

**Table S2. Design of Experiment 2: Effect of HVT-vaccination on transmission of three strains of MDV**

| **Challenge virus** | **Room** | **HVT Fc126-vaccinated**^(a)^ | **Unvaccinated**^(b)^ |
| --- | --- | --- | --- |
| 675A (vv+MDV) | 1 | Group 1A  10 infected + 10 sentinel chicks | Group 1B  10 infected + 10 sentinel chicks |
| 675A (vv+MDV) | 4 | Group 4A  10 infected & no sentinel chicks | Group 4B  10 infected & no sentinel chicks |
| 595 (vvMDV) | 2 | Group 2A  10 infected + 10 sentinel chicks | Group 2B  10 infected + 10 sentinel chicks |
| Md5 (vvMDV) | 3 | Group 3A  10 infected + 10 sentinel chicks | Group 3B  10 infected + 10 sentinel chicks |

^(a)^ Infected chickens were HVT-vaccinated, sentinel chickens were unvaccinated

^(b)^ Both infected and sentinel chickens were unvaccinated

Each of the eight groups was housed in a separate isolator
