# Supplementary material for: Imperfect Vaccination Can Enhance the Transmission of Highly Virulent Pathogens
Source: PLoS Biol. 2015 Jul 27;13(7):e1002198. doi: 10.1371/journal.pbio.1002198 (PMC4516275; doi:10.1371/journal.pbio.1002198)
Supplement: S3 Table — (DOCX) [file pbio.1002198.s008.docx]

**Table S3. Design of Experiment 3: Effect of maternally-derived antibody on shedding and transmission of two strains of MDV**

| **Challenge virus** | **Purpose of group** | **MtAb- ^(a)^** | **MtAb+ ^(b)^** |
| --- | --- | --- | --- |
| 675A (vv+MDV) | To examine transmission of MDV to in-contact sentinels | Group 1A  10 infected + 10 sentinel | Group 2A  10 infected + 10 sentinel |
|  | Collection of dust to measure shed MDV by qPCR | Group 1B  10 infected | Group 2B  10 infected |
| HPRS-B14 (vMDV) | To examine transmission of MDV to in-contact sentinels | Group 3A  10 infected + 10 sentinel | Group 4A  10 infected + 10 sentinel |
|  | Collection of dust to measure shed MDV by qPCR | Group 3B  10 infected | Group 4B  10 infected |

^(a)^ Both infected and sentinel chickens were MtAb-

^(b)^ Both infected and sentinel chickens were MtAb+
