# Supplementary material for: Imperfect Vaccination Can Enhance the Transmission of Highly Virulent Pathogens
Source: PLoS Biol. 2015 Jul 27;13(7):e1002198. doi: 10.1371/journal.pbio.1002198 (PMC4516275; doi:10.1371/journal.pbio.1002198)
Supplement: S4 Table — (DOCX) [file pbio.1002198.s009.docx]

**Table S4. Design of Experiments 4a and 4b: Transmission of MDV strain 675A in commercial maternal antibody positive HVT-vaccinated birds.**

|  | **Housing details** | **HVT FC126 Vaccinated** | **Unvaccinated** |
| --- | --- | --- | --- |
| **Experiment 4a** | Housed in 2 isolators, each containing 5 birds from Group 1 and 5 birds from Group 2.  Moved to floor pens at 4w of age where housed in previously mixed groups in separate pens within the same room | Group 1  10 birds infected with 675A (vv+MDV) | Group 2  10 sentinel birds |
|  | Housed in 2 isolators, each containing 5 birds from Group 3 and 5 birds from Group 4  Moved to floor pens at 4w of age where housed in previously mixed groups in separate pens within the same room | Group 3  10 birds infected with 675A (vv+MDV)  Group 4  10 sentinel birds |  |
| **Experiment 4b** | Housed in a floor pen with all birds mixing from 1 day of age | Group 5  10 birds infected with 675A (vv+MDV) | Group 6  10 sentinel birds |
|  | Housed in a floor pen with all birds mixing from 1 day of age | Group 7  10 birds infected with 675A (vv+) MDV  Group 8  10 sentinel birds |  |
